# Misinterpretations of the p-value in psychological research: Implications for mental health and psychological science

**DOI:** 10.1371/journal.pmen.0000242

**Published:** 2025-02-25

**Authors:** Heury Ferr

**Affiliations:** ICB (Biological Sciences Institute), Federal University of Goiás, UFG, Goiânia-GO, Brazil; PLOS: Public Library of Science, UNITED KINGDOM OF GREAT BRITAIN AND NORTHERN IRELAND

The p-value, introduced by Fisher in the early 20th century [1], remains a dominant feature of null hypothesis significance testing (NHST) in psychological research. Despite its utility as a measure of data compatibility with a null hypothesis, its misinterpretation has fostered flawed practices that undermine the reliability of scientific findings. This issue has far-reaching consequences in psychology, including within mental health research, where statistical errors can affect therapeutic practices and policy decisions. Misinterpretations of p-values perpetuate overconfidence in research findings, often leading to oversights in clinical trials, misallocation of resources, and misguided interventions in mental health [2].

Historical Roots of Misinterpretations Fisher’s original intent for the p-value was as a heuristic tool rather than a definitive decision-making criterion [1]. Its integration with Neyman-Pearson’s decision framework in the mid-20th century created conflicting interpretations. While Fisher viewed the p-value as a continuum of evidence, Neyman-Pearson’s dichotomous thresholds for decision-making fostered binary thinking [3]. This historical duality has left researchers with a fractured understanding of statistical significance, particularly in psychology, where education often overlooks these nuances. Misinterpretations have thus become embedded in research culture, exacerbated by pressures to produce significant findings [4].

## Common misinterpretations

1. **Equating p-values with Null Hypothesis Probability**: A prevalent misconception is treating the p-value as the probability that the null hypothesis is true. This error conflates frequentist and Bayesian principles, leading to misguided confidence in rejecting or accepting hypotheses [5]. This misinterpretation is particularly problematic in mental health research [6], where evidence is often used to justify treatments and interventions.
2. **Non-Significance as Evidence for the Null**: Another issue is interpreting non-significant results as support for the null hypothesis. Inadequate statistical power and small sample sizes, common in psychological and mental health studies, exacerbate this problem [7, 8]. Such errors can lead to dismissing potentially meaningful effects, hindering progress in areas like psychotherapy research or psychopharmacology [9].
3. **Overreliance on Arbitrary Thresholds**: The fixation on p < 0.05 as a criterion for significance ignores the complexity of statistical evidence. This binary approach, while simplifying decision-making, neglects the practical significance of findings [10]. In mental health, this can result in the overemphasis of marginally significant results and the dismissal of trends that might warrant further investigation [11].

Psychological Implications of Misuse The replication crisis in psychology is deeply tied to the misuse of p-values. Studies show that only a fraction of psychological findings replicate successfully, raising questions about the reliability of the field [12]. In mental health research, this issue has direct consequences for clinical practices. For example, overconfidence in statistically significant results has led to the adoption of treatments later found to be ineffective or harmful [13]. Misinterpretations of statistical evidence can also fuel public mistrust in psychological science, undermining the credibility of interventions designed to address mental health crises [14]. Below are two situations as examples, illustrating the impact of misusing p-values in mental health studies.

## Example 1: misinterpreting non-significant results

A study on the effectiveness of a new psychosocial intervention for treating depression compared two groups: one receiving the experimental intervention and the other undergoing cognitive-behavioral therapy (CBT). The study reported a p-value of 0.08 for the difference between the groups, *leading researchers to conclude that the new intervention was ineffective since the p-value exceeded the 0.05 threshold*. This interpretation ignored the fact that the study was underpowered (with low statistical power) and that the experimental intervention showed a clinically significant effect (Cohen’s d = 0.45). By interpreting the p-value as evidence of no effect, the researchers overlooked a promising intervention. As a result, funding for the approach was cut, and patients were deprived of a potentially beneficial alternative therapy.

A study investigated the impact of short guided meditation sessions on anxiety symptoms in university students. With a large sample size (n = 500), *the p-value for the difference between the experimental and control groups was highly significant (p < 0.001), suggesting that meditation reduced anxiety. However, the effect size was minimal (Cohen’s d = 0.10), indicating only a 1.5-point reduction on a 100-point anxiety scale*..

The results were widely publicized as evidence that guided meditation effectively reduced anxiety. Educational institutions and mental health programs began promoting the practice as a key solution, diverting resources from other more robust interventions, such as cognitive-behavioral therapy or mindfulness-based stress reduction programs, which had shown significantly greater effects in prior studies. These examples highlight how the misuse of p-values can lead to flawed decisions in research, clinical practice, and public policies, ultimately harming patients and wasting valuable resources.

## Impact

The impact of p-value misuse is evident in mental health studies. For example, trials investigating antidepressants have faced criticism for overinterpreting statistically significant results while neglecting small effect sizes [8]. Similarly, studies on cognitive-behavioral therapy often report p-values without contextualizing findings through confidence intervals, leaving clinicians without a clear understanding of treatment efficacy [5]. Addressing these issues requires integrating robust statistical practices into the research process.

Health Psychology and Public Health Misuse of p-values has implications beyond academic research, extending to public health and health psychology. Policy decisions often rely on studies that prioritize statistical significance over methodological rigor [15]. In the context of mental health, this can lead to the implementation of large-scale interventions based on weak evidence, diverting resources from more effective strategies. For instance, community mental health initiatives often depend on psychological studies to justify their frameworks. Misinterpreted p-values can skew these frameworks, leading to suboptimal outcomes for vulnerable populations [16].

## Educational deficits

A major contributor to the misuse of p-values is inadequate statistical education among psychologists. Many researchers lack a deep understanding of the underlying principles of NHST, perpetuating errors in study design, data analysis, and interpretation [17]. Training programs often emphasize mechanical application of tests over conceptual understanding. Addressing this deficit requires reforming educational curricula to include Bayesian statistics, effect sizes, and confidence intervals as central components of data interpretation [18].

## Solutions and alternatives

1. **Effect Sizes and Confidence Intervals**: Emphasizing these metrics over binary significance can provide a clearer picture of the practical importance of findings [9]. For example, reporting effect sizes in psychotherapy trials can help clinicians assess the real-world impact of treatments.
2. **Bayesian Methods**: Bayesian approaches offer a probabilistic framework that addresses many limitations of NHST, allowing researchers to incorporate prior knowledge and better quantify evidence [15]. These methods are particularly relevant in mental health research, where prior data often exist but remain underutilized.
3. **Reforming Publication Practices**: Journals should prioritize methodological rigor and transparency over statistically significant findings. Encouraging the publication of null results and preregistration of studies can reduce publication bias and enhance the reproducibility of psychological research [14].
4. **Statistical Education**: Integrating comprehensive statistical training into psychology programs is essential for addressing widespread misconceptions. Courses should focus on critical thinking and interpretation, equipping researchers with the tools to navigate complex data landscapes.

## Conclusion

The pervasive misinterpretations of p-values in psychology and mental health research underscore the need for systemic change. By shifting focus from arbitrary significance thresholds to a holistic understanding of statistical evidence, the field can enhance the reliability of its findings. Promoting transparency, methodological rigor, and statistical literacy is essential for fostering trust in psychological science and ensuring its contributions to mental health are both meaningful and impactful. The proposed reforms—from emphasizing effect sizes to adopting Bayesian methods—represent a path forward in addressing these critical issues.
